# p53-Dependent Activation of microRNA-34a in Response to Etoposide-Induced DNA Damage in Osteosarcoma Cell Lines Not Impaired by Dominant Negative p53 Expression

**DOI:** 10.1371/journal.pone.0114757

**Published:** 2014-12-09

**Authors:** Chiara Novello, Laura Pazzaglia, Amalia Conti, Irene Quattrini, Serena Pollino, Paola Perego, Piero Picci, Maria Serena Benassi

**Affiliations:** 1 Laboratory of Experimental Oncology, Rizzoli Orthopaedic Institute, Bologna, Italy; 2 Centre for Molecular and Translational Oncology & Department of Life Sciences- University of Parma, Parma, Italy; 3 Molecular Pharmacology Unit, Fondazione IRCCS Istituto Nazionale dei Tumori, Milan, Italy; University of Navarra, Spain

## Abstract

Osteosarcoma (OS) is the most common primary malignant bone tumor and prevalently occurs in the second decade of life. Etoposide, a chemotherapeutic agent used in combined treatments of recurrent human OS, belongs to the topoisomerase inhibitor family and causes DNA breakage. In this study we evaluated the cascade of events determined by etoposide-induced DNA damage in OS cell lines with different p53 status focusing on methylation status and expression of miR-34a that modulate tumor cell growth and cell cycle progression. Wild-type p53 U2-OS cells and U2-OS cells expressing dominant-negative form of p53 (U2- OS175) were more sensitive to etoposide than p53-deficient MG63 and Saos-2 cells, showing increased levels of unmethylated miR-34a, reduced expression of CDK4 and cell cycle arrest in G1 phase. In contrast, MG63 and Saos-2 cell lines presented aberrant methylation of miR-34a promoter gene with no miR-34a induction after etoposide treatment, underlining the close connection between p53 expression and miR-34a methylation status. Consistently, in p53siRNA transfected U2-OS cells we observed loss of miR-34a induction after etoposide exposure associated with a partial gain of gene methylation and cell cycle progress towards G2/M phase. Our results suggest that the open and unmethylated conformation of the miR-34a gene may be regulated by p53 able to bind the gene promoter. In conclusion, cell response to etoposide-induced DNA damage was not compromised in cells with dominant-negative p53 expression.

## Introduction

Human osteosarcoma (OS) is a bone tumor composed of a mass of malignant spindle cells that produce osteoid and bone. All bones can be affected, but the most involved is the metaphyseal region of long bones. OS has a bimodal age distribution with peak ages at 10–25 years and over 60. OS accounts for approximately 60% of malignant bone tumors in the first 2 decades of life [1]. OS is characterized by multiple genetic risk factors, including groups of genes or gene families involved in cell cycle control, cell proliferation or associated with cell inability to repair DNA damage [2]. At molecular level, OS cells present a great heterogeneity with multiple chromosomal abnormalities that provide a complex karyotype in more than 70% of cases, with a different response to treatment depending on genetic background [3]. Management of OS is complex and includes a variety of pre- and postoperative chemotherapeutic combinations. Doxorubicin and cisplatin are frequently used as basis of treatment and combinations with methotrexate and/or ifosfamide have demonstrated to provide additional benefits. For recurrent OS there is no accepted standard regimen and recommended chemotherapy often includes cyclophosphamide, etoposide and carboplatin [4], [5]. Etoposide, a semisynthetic epipodophyllotoxin derivate, is an agent that targets and inhibits DNA topoisomerase II (TopoII). In detail, etoposide increases TopoII-mediated DNA breakage by inhibiting the ability of the enzyme to relegate cleaved nucleic acid molecules [6], [7].

In response to DNA damage induced by etoposide, cells accumulate DNA double strand breaks (DSBs) which are identified at cell cycle checkpoints. Induction of DSBs has been considered the key mechanism responsible for etoposide pro-apoptotic and antitumor properties by increasing p53 phosphorylation (p-p53) [8]. The oncosuppressor gene TP53, located at chromosome region 17p13, is altered in ∼50% of OS [9]. TP53 is at the center of a complex molecular regulatory network and induces cell cycle arrest and apoptosis through transactivation of a variety of genes including microRNAs (miRNAs). MiRNAs are endogenous non-coding RNAs of 19–24 nucleotides that play a crucial role as post- transcriptional regulators. These small RNAs post-transcriptionally repress gene expression by recognizing complementary target sites, more often in the 3′ untranslated region (UTR) of target messenger RNAs (mRNAs). Each miRNA targets several hundreds of transcripts and it is estimated that up to 30% of human genes are regulated by miRNAs. This consideration makes miRNAs one of the largest families of genome regulators [10], [11]. MiR-34s form an evolutionary conserved miRNA family that comprises three processed miRNAs encoded by two different genes, miR-34a and miR-34b/c which are targets of p53 [12]. MiR-34a is located at chromosome region 1p36, a non-coding region located around ∼30 kb downstream of the predicted p53-binding site. Previous studies widely validated the action of p53 on the target miR-34a using a primer for pri-miR and for pre-miR-34 as well as for mature miR-34 [13], [14]. These results showed the effects of p53-dependent miR-34a activity on several candidate targets involved in cell proliferation, apoptosis and cell cycle progression such as cMET, Bcl-2, E2F3/5 and cyclin-dependent kinase 4/6 (CDK4/6). Deletion and methylation of promoter CpG islands are the most common causes for miR-34a gene silencing in tumors [15], [16]. In particular, it has been reported that especially in early neoplastic development, deregulated epigenetic modifications are as significant as genetic mutations in driving cancer development and growth [17]. DNA methylation is a post-DNA synthesis event that plays an essential role in the regulation of gene expression and chromatin organization. Methylated CpG islands within gene promoter regions present a dense and compact structure that represses promoter activity leading to gene expression loss [18], [19]. In this study we verified the response of OS cell lines with different p53 status to etoposide-induced DNA damage focusing on methylation status and expression of mature miR-34a that control downstream cell cycle pathway. In particular, we demonstrated that p53-dependent ability of etoposide to modulate mature miR-34a expression was not compromised by p53 protein with dominant negative mutation.

## Materials and Methods

### 2.1 CELL lines

Human osteosarcoma cell lines, wild-type (wt) p53 U2-OS, mutant-p53 MG63, harboring a rearrangement in intron 1 (p53−/−), and p53-null Saos-2 that present a total deletion of the sequence, were obtained from the American Type Culture Collection (ATCC) (Manassas, VA, USA). U2-OS175 and U2-OS/e cells were obtained at Istituto Nazionale Tumori, Milano, by transfection of parental U2-OS with a vector containing a mutant-p53 cDNA at site 175 (CGC Arg - CAC His) or the empty vector as previously described [20]. All cell lines were cultured in IMDM (Iscove’s Modified Dulbecco’s Medium) supplemented with 10% FBS (Fetal Bovine Serum), 2 mM L- glutamine, 100 U/ml Penicillin and 100 µg/ml Streptomycin at 37°C in a 5% CO_2_ humidified incubator and trypsinized when confluent. All in vitro experiments were independently repeated three times.

### 2.2 Small interfering RNA duplex and transfection

A small interfering RNA (siRNA) duplex targeting p53 (TP53 Validated StealthTM, Invitrogen Paisley, UK) was used in U2-OS cell line. Cells were seeded in 6-well plates (150000 cells/well) and transfected 24 h later for 5 h with specific siRNA or control siRNA (Ctrl) (Stealth siRNA Negative Control Duplex) using Lipofectamine 2000 (Invitrogen-Life Technology, Paisley, UK) according to the manufacture’s protocol.

After transfection, medium was replaced with fresh medium IMDM (Iscove’s Modified Dulbecco’s Medium) supplemented with 10% FBS (Fetal Bovine Serum) without or with increasing doses of VP16. Efficiency of down-regulation was monitored by analysis of p53 level using FACScan flow cytometer (Becton Dickinson, San Jose, CA, USA).

### 2.3 Treatment and growth-inhibition assay

OS cell sensitivity to etoposide Teva VP16 (Teva Pharma, Utrecht, Holland) was assessed by growth-inhibition assay using trypan blue to estimate the percentage of growth inhibition. All cell lines were plated at 1.5×10^5^ per well in 6-well plates allowed to attach overnight and incubated with increasing concentrations of etoposide (from 0.0005 µg/ml to 5 µg/ml). IC_50_ values, defined as concentration of drug inhibiting cell growth by 50%, were calculated for experiments with 48 h of treatment for U2-OS p53siRNA and 72 h for the other cell lines. The data were presented as mean ± SE from three independent experiments. Statistical significance was analysed by the Student’s t-test and a probability value of p≤0.05 was considered to indicate a statistically significant difference.

### 2.4 RNA extraction and miR-34a expression analysis by real time PCR

Total RNA was extracted from cell lines before and after 24 h–48 h of exposure to etoposide IC_50_ using TRIzol Reagent (Invitrogen, Carlsbad, CA, USA) according to the manufacturer’s protocol and stored at 80°C in RNAsecure reagent (Ambion, Inc, Austin, TX, USA). Concentration of total RNA was measured with spectrophotometer, purity and quality were checked by a denatured gel electrophoresis. Reverse transcription and RealTime PCR (RT-PCR) were carried out following TaqMan MicroRNA Assay Protocol (Applied Biosystems, Life Technology) and the expression of miR-34a (miRNA Assay n.000426) were quantified using ΔCT comparative method (Applied Biosystems, User Bulletin N°2 P/N 4303859) and normalized using RNU44 as endogenous reference (miRNA Assay n°.001094). The data were presented as mean ± SE from three independent experiments.

### 2.5 Methylation-specific polymerase chain reaction (MSP)

DNA was extracted from OS cell lines by standard method. DNA was treated with bisulfite by EpiTect Bisulfite Kit (Quiagen, Hilden, Germany) to determine aberrant miR-34a promoter methylation status. The procedure comprised different steps: bisulfite-mediated conversion of unmethylated cytosines; purification and elution of DNA and finally amplification of purified DNA by polymerase chain reaction (PCR). Primers used for methylated methylation-specific polymerase chain reaction (M-MSP) and unmethylated methylation-specific polymerase chain reaction (U-MSP) designed for the CpG area upstream of the miR-34a promoter: U-MSP 34a Reverse: 5′CAA-ACA-AAA-CAC-ATA-AAA-ACA-ACA-3′, U-MSP 34a Forward: 5′GGG-GAT-GAG-GAT-TAG-GAT-TTT-3′, M-MSP 34a Reverse: 5′ACA-AAA-CGC-ATA-AAA-ACG-ACG-3′, M-MSP 34a Forward: 5′GGG-GAT-GAG-GAT-TAG-GAT-TTC-3′. PCR products were analyzed on 2% agarose gel.

### 2.6 Chromatin Immunoprecipitation (ChIP) assay

DNA and protein complexes were reversibly cross-linked in living cells by adding formaldehyde directly to cell culture medium at 1% final concentration to maintain the association of proteins with their target DNA sequence. Chromatin extract was then shared by sonication to DNA fragments with an average size of 200–1000 bps, cleared by centrifugation with the addiction of sonicated salmon sperm DNA/protein A agarose (Millipore, Temecula, CA). Precleared chromatin was incubated overnight at 4°C on rotating plate with anti-p53 (aa20-25) dilution:1∶1000 (AbD Serotec, Kidlington, OX5 1GE, UK). Precipitation continued with the addition of salmon sperm DNA/protein A agarose. Precipitates were washed sequentially under stringent condition to remove un-specifically bound chromatin and were eluted. Cross-links were reversed, proteins were digested and ChiP DNA purified. DNA sequences associated with precipitated protein were identified by PCR using 2 µL of immunoprecipitated DNA and promoter-specific primers (Invitrogen, Carlsbad, CA, USA) for miR-34a promoter sequence containing p53 cis-elements (Forward: 5′-TTT TCA GGT GGA GGA GAT GC-3′, Reverse: 5′-CAG GAC TCC CGC AAA ATC T-3′). Immunoprecipitated DNA with non-specific immunoglobulins (IgG; Santa Cruz Biotechnology, Santa Cruz, Ca, USA) was considered as negative control. PCR products were run on 2% agarose gel and visualized.

### 2.7 Cell cycle analysis

OS cells were plated overnight at 1.5×10^5^ cells per well in 6-well plates and cell cycle distribution analysis was performed before and after 24–48 h exposure to etoposide concentration corresponding to IC_50_. After trypsinization and fixation with 70% ethanol, cells were stained for total DNA content with a solution containing 20 µg/ml propidium iodide. Cell cycle distribution was then analyzed with a FACScan flow cytometer (Becton Dickinson). Cell fraction percentage was presented as mean from three independent experiments.

### 2.8 Apoptosis measurement

Apoptotic cell death was analyzed with Annexin V-FITC apoptosis detection kit (MEBCYTO Apoptosis kit, MBL International, Woburn, MA, USA). The green (FL1) and red (FL2) fluorescence of Annexin/propidium iodide (PI)-stained live cells and PI-stained fixed cells was analyzed with a FACSCalibur flow cytometer and CellQuest Software (BD Biosciences, San Jose, CA, USA), using a peak fluorescence gate to exclude cell aggregates. According to protocol, after 24 h and 48 h from transfection, adherent cells were briefly trypsinized and re-suspended in 500 µl staining solution containing FITC-conjugated Annexin V antibody and PI. After incubation, cells were analyzed by flow cytometry. Basal apoptosis and necrosis were identically determined on untreated cells using the same procedure. Data were presented as mean ± SE from three independent experiments.

### 2.9 Co-immunoprecipitation and western blot analysis

According to standard procedures, 300 µg of OS cell lysate were immunoprecipitated with antibodies anti-p-p53 (hSer20) (Santa Cruz Biotechnology) and anti-p53(aa20-25) (AbD Serotec, Kidlington, OX5 1GE, UK), fractioned by 8% SDS-polyacrylamide gel and transferred to nitrocellulose membranes. Western blot analysis was performed by using anti-p-p53(hSer20) (dilution 1∶200; Santa Cruz Biotechnology) and anti-p53 (aa20-25) (dilution 1∶1000; AbD Serotec). Expression levels of total CDK4, cyclin D1 and CDK4 bound to cyclin D1 were determined before and after 48 h exposure to etoposide concentration corresponding to IC_50_. 250 µg of cell lysate were immunoprecipitated with 10 µl of antibodies to CDK4 (C-22) (Santa Cruz Biotechnology) and cyclin D1 (Cell Signaling Technology, Beverly, MA, USA) adding Gamma Binding Plus Sepharose. Precipitates were analysed by 10% SDS-polyacrylamide gel followed by western blot with anti-CDK4 (dilution 1∶200; Santa Cruz Biotechnology) and anti-cyclin D1 (dilution 1∶100; Cell Signaling Technology). Western blot with CDK4 antibody was used in cyclin D1 immunoprecipitates to analyse CDK4 bound to D1. Anti-actin (dilution 1∶100000; EMD Millipore Corporation, Billerica, MA, USA) was used as control and horseradish peroxidase-conjugated anti-rabbit IgG or anti-mouse IgG were used as secondary antibodies. The signal was visualized by Supersignal West Pico Chemiluminiscent Substrate (Pierce, Rockford, IL, USA) and quantified by GS-670 imaging densitometer (Bio- Rad, Hercules, CA, USA).

## Results

### 3.1 Levels of p53 in cell lines with different p53 status

**Figure 1:**
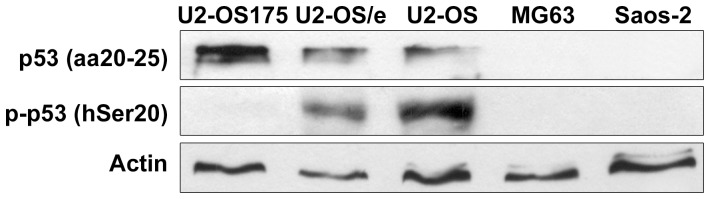
p53 protein expression in OS cells. wt-p53 U2-OS, U2-OS transfected with empty vector (U2-OS/e) and p53-impaired U2-OS175 cells were positive to anti-p53 that binds the transactivation site of N-terminal domain (aa20-25), with increased expression in U2-OS175 cells. U2-OS and U2-OS/e also presented accumulation of p53 phosphorylated at Ser20 residue (p-p53). MG63 and Saos-2 were negative to both antibodies. Actina was used as loading control.

p53 expression in OS cell lines was assessed using anti-p53 (aa20-25) antibody that binds the transactivation site of N-terminal domain of p53 protein and recognizes both wild type and mutant forms and anti-p-p53 (hSer20) antibody that recognizes p53 phosphorylated form at Ser20 residue. Western blot analysis with anti-p53 (aa20-25) confirmed expression of p53 protein in wt-p53 U2-OS as well as in U2-OS transfected with empty vector (U2-OS/e). U2-OS transfected with mutant-p53 cDNA at site 175 (U2-OS175) presented increased p53 expression compared to both (Fig. 1). However, only U2-OS and U2-OS/e cell lines presented an accumulation of p53 phosphorylated form at the residue hSer20 indicating the presence of a stable and functional protein whereas U2-OS175 cell line was negative (Fig. 1). OS cell lines with mut-p53 (−/−) (MG63) and p53-nul (Saos-2), identified as “p53-deficient”, resulted negative to both antibodies (Fig. 1).

### 3.2 Etoposide inhibits viability of OS cells

**Figure 2:**
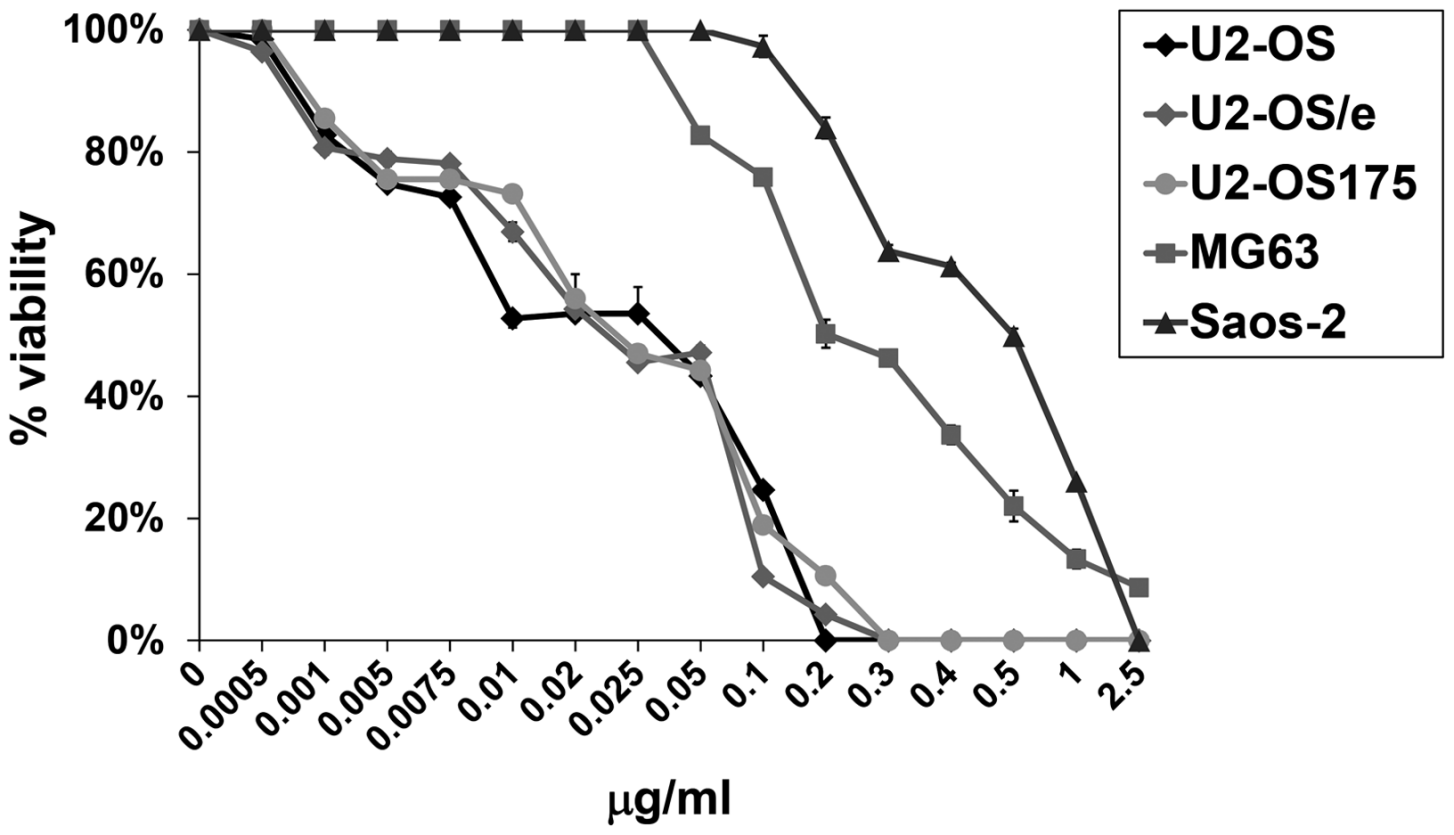
Growth-inhibition assay. U2-OS and U2-OS175 cells showed a similar viability Trend with higher sensitivity to etoposide at 72 h than MG63 and Saos-2 cells. No differences between U2- OS and U2-OS/e were observed. Data were presented as mean ± SE from three independent experiments. Student’s test indicated significantly lower IC_50_ mean values at 72 h of treatment in U2-OS, U2-OS/e and in U2-OS175 than in p53-deficient Saos-2 and MG63 ((p<0.05).

Susceptibility of OS cells to increasing concentrations of etoposide was assessed by growth-inhibition assay that showed a similar trend of drug-response in U2-OS and U2-OS/e cells as well as in U2-OS175 cells expressing dominant-negative form of p53 (Fig. 2). Cell counting indicated that these cell lines were more sensitive to etoposide with significantly lower IC_50_ mean values at 72 h treatment (0.03±0.016 µg/ml, 0.03±0.012 µg/ml and 0.02±0.01 µg/ml respectively) than p53-deficient Saos-2 and MG63 (0.5 µg/ml ±0.041and 0.2±0.013 µg/ml respectively) (p<0.05).

### 3.3 Induction of miR-34a expression level

**Figure 3:**
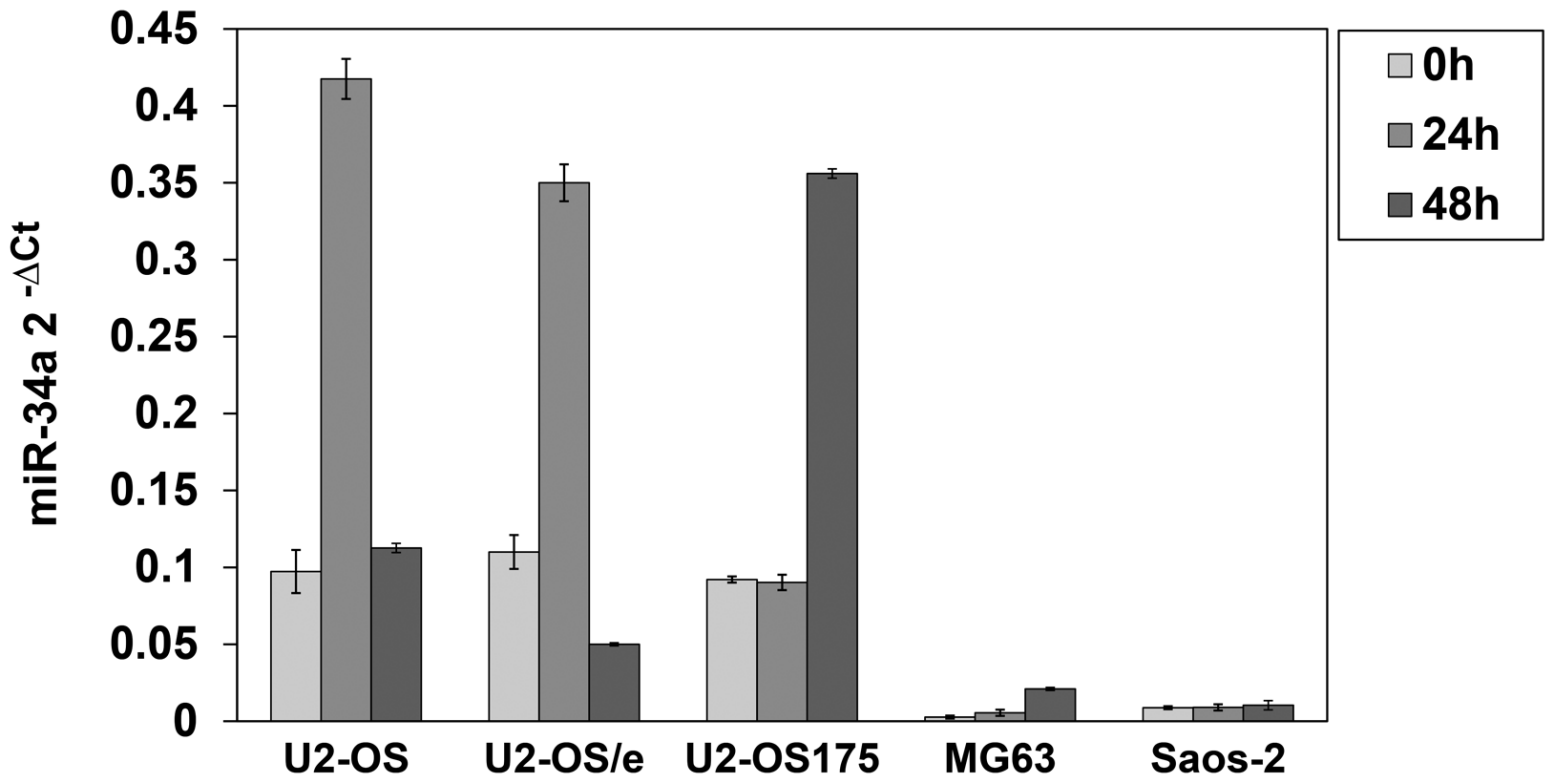
RT-PCR analysis of miR-34a. Increased expression of miR-34a was seen in U2-OS, U2-OS/e and in U2-OS175 in response to etoposide treatment at 24 h and 48 h respectively. No relevant changes were evident in p53-deficient MG63 and Saos-2, also showing lower basal miR-34a levels. Data were presented as mean ± SE from three independent experiments.

When OS cells were treated with respective IC_50_ concentrations of etoposide, induction of miR-34a gene expression was evaluated by RT-PCR. Mature mir-34a basal levels expressed as 2^−ΔCT^ were lower in p53-deficient than in U2-OS and U2-OS175 (Fig. 3). In U2-OS and U2-OS/e cells respectively 4.0-fold and 3.2-fold increase of miR-34a levels was seen at 24 h drug exposure. However, at 48 h the expression shifted towards control levels. A noticeable increase of miR-34a level (3.9-fold) was seen at 48 h in U2-OS175 cells while in MG63 and Saos-2 responded with a less relevant increased expression of 2.6-fold and 1.2-fold respectively. (Fig. 3).

### 3.4 Promoter methylation of miR-34a gene

**Figure 4:**
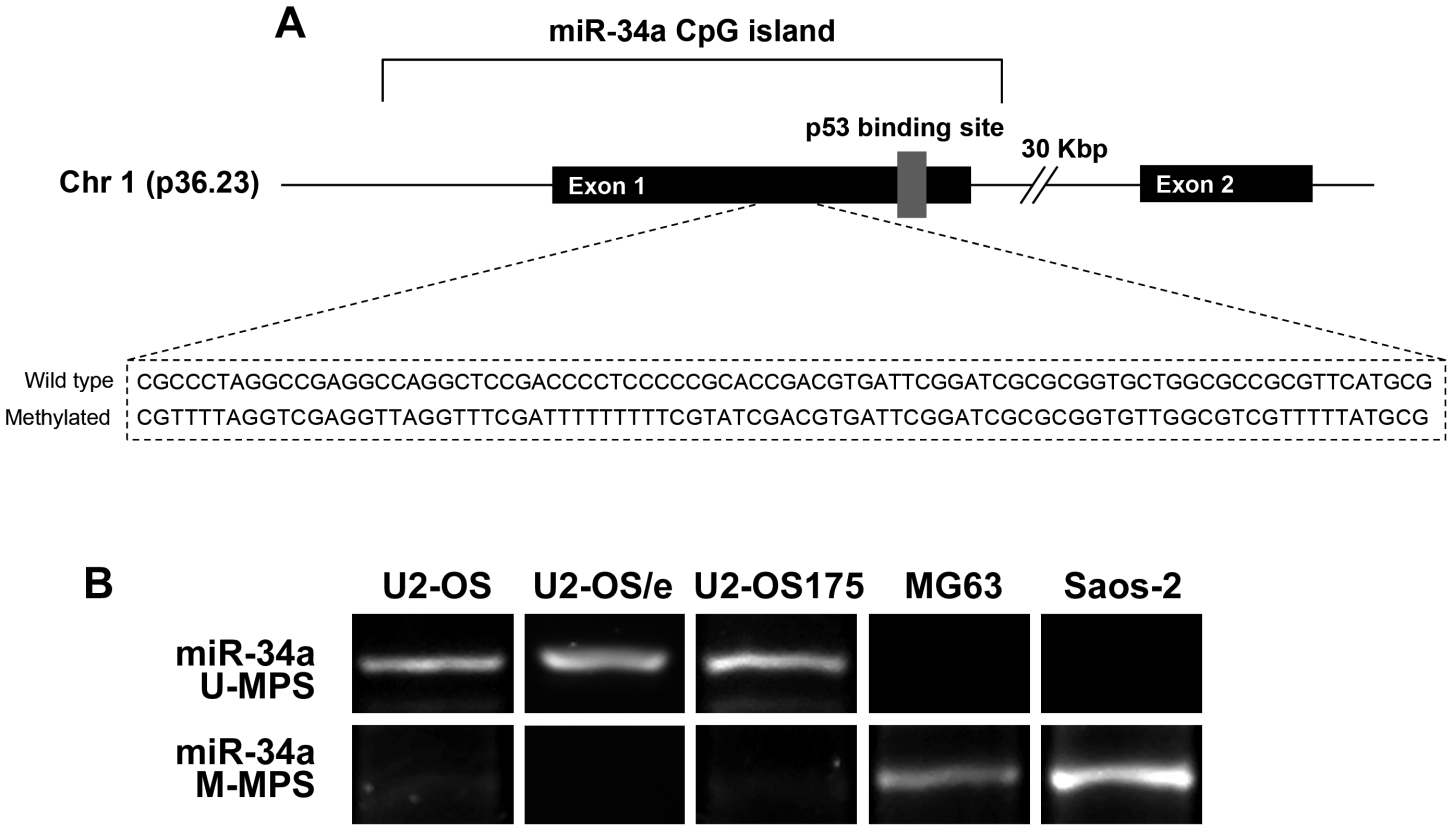
miR-34a gene genomic organization and methylation specific PCR. (**A**) The position of p53 binding site and primers for wild-type and methylation sequences on CpG region are indicated. (**B**) After bisulphite treatment, U2-OS, U2-OS/e and U2-OS175 showed complete unmethylation (U-MPS) of miR-34a promoter; in contrast MG63 and Saos-2 cells presented methylation (M-MPS) of both alleles.

Since epigenetic down-regulation by CpG methylation is commonly seen in tumor cells, we studied methylation status of miR-34a in the genomic region upstream of the p53 binding site (Fig. 4A).

After bisulphite treatment, MSP showed an aberrant methylation (M-MSP) of miR-34a CpG islands in both MG63 and Saos-2. Conversely, CpG islands of miR-34a were completely unmethylated (U-MPS) in U2-OS, U2-OS/e and in U2OS175 cells (Fig. 4B), stressing the relationship between gene open conformation and p53 protein expression.

### 3.5 Chromatin Immunoprecipitation (ChIP) assay

**Figure 5:**
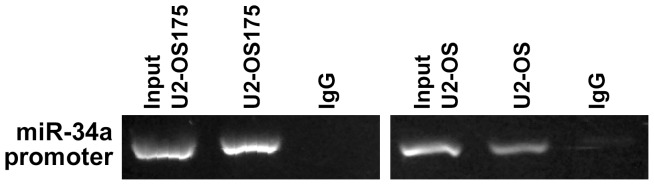
Chromatin Immunoprecipitation assay (ChIP). Interaction between p53 and miR-34a promoter was present in both U2-OS and U2-OS175. *INPUT = positive control; IgG = negative control.*

To verify that the ability of p53 protein to bind the promoter of miR-34a target gene is not compromised by mutation at site 175 we performed ChIP assay. The analysis showed binding between p53 and the promoter of miR-34a in U2-OS and U2-OS175 cells (Fig. 5), but not in the p53-deficient cell lines, MG63 and Saos-2 suggesting that increase of miR-34a expression in response to etoposide was dependent on p53 recruitment and not impaired by expression of dominant negative p53.

### 3.6 Cell cycle distribution and co-immunoprecipitation

**Figure 6:**
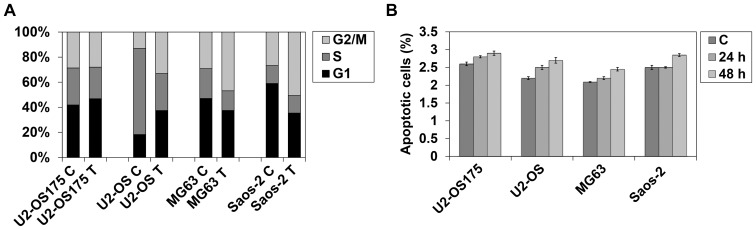
Cell cycle analysis and apoptosis. (**A**) After 48 h of etoposide treatment (T), BrDU incorporation showed cell accumulation in G1 phase in U2-OS and U2-OS175 cells and in G2/M phase in MG63 and Saos-2 when compared to untreated cells. (**B**) By Annexin V-FITC assay, no significant increase of apoptotic cells was observed in OS cell lines after 24 h and 48 h of treatment. Data were presented as mean ± SE from three independent experiments. *C = Untreated cells.*

After 48 h exposure to IC_50_ etoposide, BrDU incorporation showed a different cell cycle distribution in OS cell lines (Fig. 6A). U2-OS cells presented cell accumulation in G1 phase, (from 18.3% of total cell populations in non-treated to 37.4% in treated cells) and G2/M (from 13.0% to 33.0%) accompanied by a relevant cell decrease in S phase (from 68.6% to 29.5%). Although a reduced G1 accumulation in U2-OS175 cells was expected, given the expression of dominant negative p53, slight changes in cell cycle distribution were seen after etoposide treatment (from 41.8% to 46.8% in G1; from 28.6% to 27.9% in G2/M and from 29.4% to 25.1% in S phase). No significant differences were observed between U2-OS and U2-OS/e cells. p53-deficient MG63 responded to etoposide with a marked accumulation of G2/M (from 29.1% to 46.8%), concomitant with pronounced cell decrease in G1 (from 47.0% to 37.5%) and S phase (from 23.7% to 15.6%). Similarly, in Saos-2 cells, etoposide-induced cell accumulation in G2/M (from 26.6% to 50.5%) accompanied by a strong decrease in G1 (from 58.9% to 35.3%). Surprisingly, a very slight and non significant increase in percentage of apoptotic cells was observed using Annexin V-FITC assay in all OS cell lines reaching the peak of 22% in U2-OS after 48 h of etoposide exposure (Fig. 6B).

**Figure 7:**
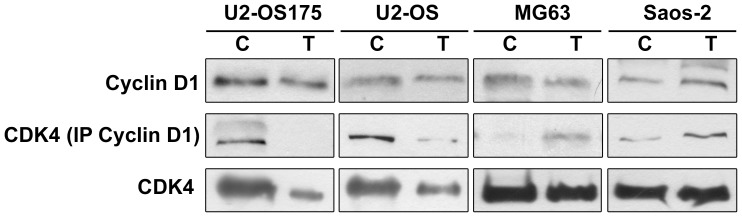
Western blot of total and D1-bound CDK4. In contrast to MG63 and Saos-2 cells, decreased levels of total CDK4 and CDK4 bound to cyclin D1 were observed in U2-OS and U2-OS175 cells after 48 h of etoposide treatment when compared to untreated cells. No differences in cyclin D1 levels were seen. Etoposide treatment did not affect CDK4 level in both MG63 and Saos-2 cell lines. A slight increase of D1-bound CDK4 was evident. *C = Untreated cells; T = Etoposide treated cells.*

Given the evidence that cell cycle deregulation depends on modulation of functional cyclin D1/CDK4 complex, we analyzed the expression of total CDK4, a target of miR-34a, and CDK4 bound to cyclin D1. Immunoblot analysis revealed lower amounts of total CDK4 after etoposide treatment in U2-OS and U2-OS175 cell lines, concomitant with a marked decrease of CDK4 bound to D1 (Fig. 7), in accordance to cell arrest in G1 phase. No differences between U2-OS and U2-OS/e were observed in cell cycle distribution and in total and D1-bound CDK4 expression (data not shown). In contrast in MG63 and Saos-2 cell lines etoposide treatment did not affect total CDK4 level but even showed a slight increase of D1-bound CDK4.

### 3.7 p53 silencing of U2-OS cells

**Figure 8:**
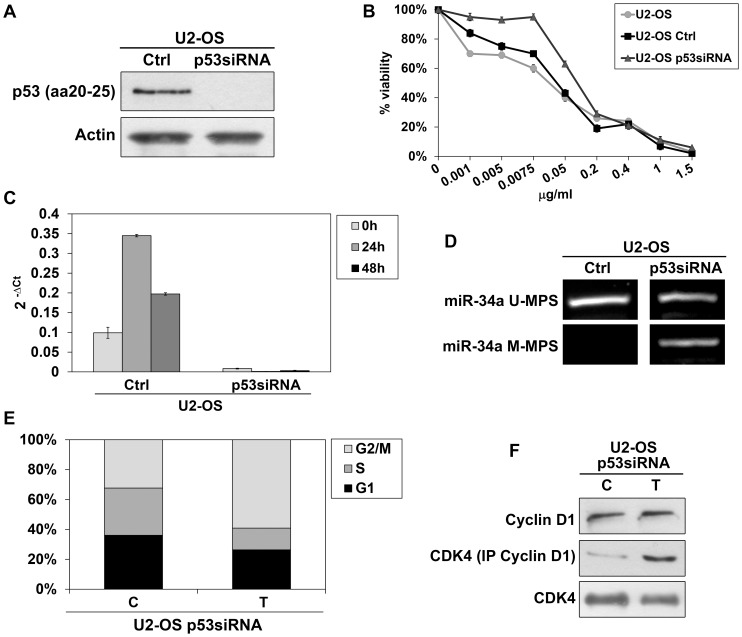
Response of p53siRNA U2-OS cells to etoposide. (**A**) Inhibition of p53 expression in p53siRNA U2-OS. Actin was used as loading control. (**B**) p53siRNA U2-OS were less sensitive to etoposide when compared with parental and Ctrl U2-OS;). Student’s test from three independent experiments indicated significantly higher IC_50_ mean values at 72 h of treatment in p53siRNA U2-OS than in Ctrl and parental U2-OS cells; p = 0.05 (**C**) Etoposide treatment did not induce mature miR-34a expression in p53siRNA U2-OS, as opposed to Ctrl U2-OS. (**D**) p53siRNA U2-OS cells presented CpG island methylation (M-MSP) of one of the two alleles of miR-34a. In Ctrl U2-OS both alleles were unmethylated. (**E**) p53siRNA transfection determined lengthening of G2/M phase after 48 h of etoposide treatment when compared to untreated cells. (**F**) Western blot of cyclin D1 and CDK4 in p53siRNA cell showed increased amount of CDK4 linked to cyclin D1 and total CDK4 after etoposide treatment when compared to control. No differences in cyclin D1 levels were seen. *Ctrl = siRNA negative control duplex; C = Untreated cells; T = Etoposide treated cells.*

To support involvement of p53 in epigenetic modification of miR-34a and in response to etoposide treatment, we used a siRNA approach in wt-p53 U2-OS cells to knockdown p53 expression (Fig. 8A). Silencing of p53 by p53siRNA transfection induced a noticeable decrease of sensitivity, with higher IC_50_ values at 72 h treatment (0.1±0.01 µg/ml) than Ctrl U2-OS and parental U2-OS cells (0.03±0.01 µg/ml) (p = 0.05) (Fig. 8B). p53siRNA U2-OS presented IC_50_ values similar to those observed in MG63 and Saos-2 cells. Moreover, p53siRNA U2-OS did not increase miR-34a expression after exposure to etoposide (Fig. 8C), but presented a partial gain of CpG island methylation (Fig. 8D), highlighting the close connection between loss of p53 expression and DNA methylation. In siRNA negative control, data were similar to parental U2-OS cells in terms of p53 expression and in terms of response to etoposide for cell viability, miR-34a expression and unmethylated status. Cell cycle analysis of p53siRNA U2-OS showed a drug-response similar to MG63 and Saos-2 cells with a marked accumulation of G2/M and cell decrease in G1 and S phase when compared to untreated cells (Fig. 8E). Accordingly, while total CDK4 level remained constant, D1-bound CDK4 presented a slight increase after etoposide exposure compared to untreated cells (Fig. 8F). This confirms CDK4 as target of miR-34a and supports its role in cell progression towards G2/M phase. No different drug-response was observed between parental and Ctrl siRNA U2- OS cells.

## Discussion

The miR-34 family has been identified as a p53 target and plays a key role as regulator of tumor suppression in many cancers controlling cell cycle arrest and apoptosis [20]–[22]. Previous studies reported that over-expression of miR-34a inhibits OS tumor growth and metastasis down-regulating c-Met [23] and that adriamycin exposure or irradiation induced miR-34a expression in wt-p53 OS cell lines, but not in nul-p53 Saos-2 [14]. In p53-mutant pancreatic cancer cells, restoration of miR-34 expression significantly inhibited cell growth inducing apoptosis and cell cycle arrest [24].

In this study, we analysed the response of osteosarcoma cells to etoposide, an antitumor drug that inhibits Topoisomerasi II catalytic activity [25]. Differently from p53-deficient cells, wt-p53 U2-OS carrying active p–p53 function and p53-impaired U2-OS175 cells lacking p–p53-Ser20 phosphorylated form, induced p53-dependent miR-34a increased expression. R175H is the most frequent p53 alteration found in cancer and affects 2 amino acid loops interacting with the minor groove of the DNA molecule. p53 protein conformational changes lead to acquisition of new oncogenic activities related to metastatic behavior [26]–[28]. IARC TP53 Database (www-p53.iarc.fr) provides somatic and germline p53 mutations and shows that the protein with missense mutation is a non-functional transcription factor. After demonstrating that miR-34a basal levels were lower in p53-deficient than in U2-OS and U2-OS175 cells, we also found that these cell lines had a higher sensitivity to etoposide than MG63 (p53^−/−^) and Saos-2 (p53-null) inducing miR-34a expression through direct binding between p53 and miR-34a gene promoter. This suggested that recruitment of p53 by miR-34 was not impaired by expression of dominant negative p53. However, the slight increase of miR-34a at 48 h of drug incubation in MG63 p53-deficient cells supported the hypothesis that other p53–independent factors may induce miR-34a expression in OS cells [14]. This interesting point could be the object of further investigation. Yan et al. [23] showed that overexpression of miR-34a significantly suppressed cell proliferation, whereas miR-34a down-regulation caused by epigenetic alterations has been found in OS and in cancer metastasis [14], [29]. By inspecting genomic region upstream of the binding site of p53 in miR-34a gene, previous studies identified a prominent methylated CpG island that caused gene silencing [30]. The deregulated mechanism of epigenetic machinery can promote tumor progression. In particular, epigenetic silencing of tumor suppressor miR-34a confers a proliferative advantage to tumor cells [17], [31]. In U2-OS and U2-OS175 cells, miR-34a promoter was unmethylated in both gene alleles, while MG63 and Saos-2 showed CpG methylation of the two alleles in accordance with very low expression levels and lack of miR-34 induction after etoposide exposure. In the p53 network, miR-34a controls the cell cycle and inhibits tumor growth through down-regulation of CDK4 [13], [14]. Accordingly, after 48 h of exposure to etoposide, U2-OS and U2-OS175 presented cell accumulation in G1 phase concomitant with a decrease in the amount of total and cyclin D1-bound CDK4 when compared to untreated cells. In contrast, MG63 and Saos-2 cells responded to etoposide with a peak of cell accumulation at G2/M phase triggering a signaling cascade that controls mitotic entry and allows repair of DNA damage [32]. p53-dependent ability of etoposide to modulate mature miR-34a expression and induce a predominant cytostatic effect through its target, CDK4, was confirmed in p53siRNA U2-OS showing a drug response similar to MG63 and Saos-2 cells. In p53siRNA U2-OS we observed lack of induction of miR-34aexpression, cell cycle progression towards G2/M phase and gain of partial methylation of the gene promoter. In conclusion, although missense mutations strongly limit overall cellular p53 function supporting dominant-negative actions [20], [33], p53-dependent etoposide sensitivity and its effect on miR-34a expression, CDK4/cyclinD1 complex level and G1 arrest was the same in both wt-p53 U2-OS and U2-OS with mutant-p53 cDNA at site 175. MG63 and Saos-2 as well as p53siRNA U2-OS were less drug sensitive and required higher concentrations of etoposide to induce a 50% decrease of cell viability. Moreover, p53-deficient cells showed arrest in G2/M phase suggesting that these cells may be sensitized to genotoxic agents through G2 checkpoint abrogation [32]. Given that miR-34a methylation occurred in MG63 and Saos-2 as well as in p53siRNA U2-OS but not in wt-p53 and p53-R175H U2-OS, our results suggest that the open and unmethylated conformation of miR-34a gene may be regulated by p53 able to bind the gene promoter.
